# Neurophysiological markers of ADHD symptoms in typically-developing children

**DOI:** 10.1038/s41598-020-80562-0

**Published:** 2020-12-31

**Authors:** Kirsten Hilger, Jona Sassenhagen, Jan Kühnhausen, Merle Reuter, Ulrike Schwarz, Caterina Gawrilow, Christian J. Fiebach

**Affiliations:** 1 Department of Psychology, Goethe University Frankfurt, Frankfurt am Main, Germany; 2 Department of Psychology I, University Würzburg, Marcusstr. 9-11, 97070 Würzburg, Germany; 3 IDeA Center for Individual Development and Adaptive Education, Frankfurt am Main, Germany; 4 LEAD Graduate School and Research Network, Eberhard Karls University Tübingen, Tübingen, Germany; 5 Department of Psychiatry, Psychosomatics and Psychotherapy in Childhood and Adolescence, University Hospital Tübingen, Tübingen, Germany; 6 Department of Psychology, University Tübingen, Tübingen, Germany; 7 Brain Imaging Center, Goethe University Frankfurt, Frankfurt am Main, Germany

**Keywords:** Attention, Cognitive control

## Abstract

Children with attention-deficit/hyperactivity disorder (ADHD) are characterized by symptoms of inattention, impulsivity, and hyperactivity. Neurophysiological correlates of ADHD include changes in the P3 component of event-related brain potentials (ERPs). Motivated by recent advances towards a more dimensional understanding of ADHD, we investigate whether ADHD-related ERP markers relate to continuous variations in attention and executive functioning also in typically-developing children. ERPs were measured while 31 school children (9–11 years) completed an adapted version of the Continuous Performance Task that additionally to inhibitory processes also isolates effects of physical stimulus salience. Children with higher levels of parent-reported ADHD symptoms did not differ in task performance, but exhibited smaller P3 amplitudes related to stimulus salience. Furthermore, ADHD symptoms were associated with the variability of neural responses over time: Children with higher levels of ADHD symptoms demonstrated lower variability in inhibition- and salience-related P3 amplitudes. No effects were observed for ERP latencies and the salience-related N2. By demonstrating that ADHD-associated neurophysiological mechanisms of inhibition and salience processing covary with attention and executive functioning in a children community sample, our study provides neurophysiological support for dimensional models of ADHD. Also, temporal variability in event-related potentials is highlighted as additional indicator of ADHD requiring further investigation.

## Introduction

Children with attention-deficit/hyperactivity disorder (ADHD) are characterized by developmentally inappropriate symptoms of inattentiveness, hyperactivity, and impulsivity^1^. ADHD is the most commonly diagnosed psychiatric disorder in children^2^, affecting 3–5% of school-aged children, and is associated with problems in social and emotional functioning, diminished academic success, and a globally increased risk for psychiatric problems^3^. Children affected by ADHD typically demonstrate poorer performance on tasks requiring response inhibition, working memory, planning, and the flexible adaptation of action and thought, which is often attributed to a core deficit in executive functions^4^ but alternatively also to more fundamental deficits related e.g., to the allocation of attentional or cognitive resources^5,6^. While many earlier studies suggest that such executive deficits differentiate properly between children with and without ADHD^7^, newer findings reveal a more heterogeneous picture, with some but not all studies reporting clear dissociations between groups of clinically affected patients and groups of typically-developing controls^8^.

ADHD has mostly been treated as categorical concept. Accordingly, investigations of behavioral and neural mechanisms underlying ADHD primarily relied on group comparisons between clinically affected patients and healthy controls. However, these approaches ignore that symptoms vary substantially between affected persons^9–12^ and even subclinical variations in ADHD symptoms significantly impact cognitive functioning and psychological wellbeing^13^. This suggests that the investigation of continuous variations in ADHD-related behavior can provide insights into whether or not neurocognitive models of ADHD (like the *default-mode interference hypothesis*^14^, or the *cognitive-energetic model*^5,6,15^) can be generalized to explain behavioral variations in attention and executive functions within the general population^16^.

Neurophysiological investigations of ADHD focused primarily on neural mechanisms of selective or sustained attention, response inhibition, effort allocation, or distractor interference^17^. Multiple variations of Stop-Signal, Go-NoGo, and Continuous Performance Tasks (CPT^18,19^) revealed differences between clinically affected patients and groups of healthy controls^5,17^. In such tasks, children with ADHD typically demonstrate higher rates of omission and commission errors and increased variability in reaction times^20^. This goes along with changes in neurophysiological correlates of these cognitive processes. One of the most established markers of ADHD is a reduced amplitude and longer latency of the inhibition-related P3 component of the event-related brain potential (ERP)^21,22^. However, these ERP effects have so far not been studied extensively in the context of a dimensional framework of ADHD, and it is therefore not yet clear how these ERP components are related to individual differences in problems in attention and executive functioning.

Recent research further suggests that children with ADHD exhibit greater distractibility to salient but task-irrelevant events^23^. Most evidence for aberrant salience processing in ADHD comes from functional MRI studies focusing on interactions between large-scale functional brain networks^23–25^ and from ERP studies using a wide variety of different experimental paradigms^26–28^. However, most of these studies focused on concepts like motivational salience^27^ or emotional salience^29^ and do not assess neurophysiological responses to ‘pure’ physical stimulus salience. Initial efforts into the direction of disentangling motivational and perceptual effects of salience were made by a recent investigation that differentiates between salience effects in the P3 linked to novelty, targetness (task-relevance), and deviancy of the stimuli within an adult ADHD sample^30^. Task-irrelevant stimulus salience also elicits inhibitory processes indexed in the ERP by anterior N2 effects^31^, but these were not modulated by ADHD in the study of Godefroid and Wiersema^30^. To the best of our knowledge, such ADHD-related effects of physical stimulus salience have so far not been studied in children.

A related but independent line of research suggests a reduction of the stability (or increase in variability) of cognitive processes as a major component of the cognitive deficits in children with ADHD^32^. Several studies found increased variability in response times over the course of an experiment and less consistent error rates in clinically affected patients when compared with groups of healthy controls^33,34^. Recently, research has begun to investigate whether potential associations between ADHD and different levels of intra-subject variability also manifest neurophysiologically. These studies focused primarily on the investigation of inhibitory processes and found evidence for higher variability in the amplitude^34–36^ and latency^35^ of inhibition-related ERP components (i.e., the P3) in ADHD. In contrast, ADHD-related variability of ERP components associated with the processing of physical stimulus salience has not yet been investigated. However, the distractibility that is typical for ADHD becomes particularly relevant in the presence of salient but task-irrelevant stimulation and is known to fluctuate over time^14^. Investigating the variability over time of neurophysiological mechanisms involved in salience processing may thus provide valuable contributions to the understanding of ADHD-related behavioral symptoms.

In the present study, we assessed the association between an integrated index of behavioral ADHD symptoms and its relationship to established neurophysiological markers of ADHD, in a subclinical sample of 9 – 11 year old school children. Based on previous research, we hypothesized that children with more problems in attention and executive functioning should show reduced P3 amplitudes (Hypothesis 1a) and longer P3 latencies (Hypothesis 1b) associated with the inhibition of a dominant task response^21,22^. Potential associations between ADHD-related behaviors and P3 and N2 effects elicited by the processing of task-irrelevant physical stimulus salience will be explored, as the available literature did not allow us to formulate specific hypotheses. The second focus of our analyses was to investigate the role of intra-individual variability of ERP components over time. Although available empirical evidence is only sparse and provides rather preliminary insights, we hypothesized that more problems in attention and executive functioning should go along with higher variability in inhibition-related P3 amplitudes (Hypothesis 2a) and latencies (Hypothesis 2b)^34–36^. Finally, we explore for the first time potential associations between individual differences in ADHD-related behaviors and the temporal variability of ERP effects (P3, N2) elicited during the processing of physical stimulus salience.

## Methods

All analysis code used in the current study has been deposited on GitHub at https://github.com/KirstenHilger/AttentionGo1. The data of the whole project will be deposit on zenodo.org after the project is completed (part of ongoing longitudinal project, see below). The data used in the actual study can be assessed from the authors upon direct request.

### Participants

The current study was conducted as part of a longitudinal project that investigates developmental trajectories of children with varying degrees of attentional and executive function problems and that focusses on temporal fluctuations in cognitive, emotional, and social behavior on various different time scales. All children were in the 5^th^ grade of the German school system, that is, in the first year of secondary school. Recruitment was conducted via local advertisements and special-organized information seminars for parents and children at participating schools. Recruitment was restricted to two out of four different school types in the German school system (i.e., “Realschule” and “Gesamtschule”) as problems in attention and executive functioning were suggested to be more frequently present among respective students. As compensation for their general participation in the project, children and their families received coupons for special events (e.g., visit in the zoo, cinema) and additional participation in the electroencephalography (EEG) recordings was reimbursed with book vouchers. The data used in the actual study was acquired during the first out of three measurement bursts. 32 children (aged 9–11 years) completed the EEG measurement. We ensured that all children had normal or corrected to normal vision and hearing abilities and were free of any neurological, psychological, or developmental disorders (beyond ADHD). Four children had a formal diagnosis of ADHD. All of those children were treated with methylphenidate (10–20 mg), which was taken early in the morning (between 7 and 8.30 am) also at the day of EEG measurement; EEG measurements took place after school in the late afternoon (3 – 7 pm). One participant was excluded due to technical problems during data acquisition, so that the final sample comprised 31 right-handed children (*M* = 10.7 years, *SD* = 0.53; 14 males; IQ measured by the Raven’s Standard Progressive Matrices, SPM^37^, German version^38^: range 71 – 133, *M* = 97.38, *SD* = 11.66). Note that the sample size of the present study was limited by the project from which subjects were recruited. A power calculation indicated that given the current sample size, only large effects (i.e., correlations > 0.48) can be detected with 80% statistical power. We therefore treat effects smaller than around *r* =  ~ 0.48 as hypothesis-generating in nature.

### Questionnaire measures

Problems in attention and executive functions were assessed with the German version of the Conners’ scales for attention and behavior (Conners 3; long version, parent rating^39^; German adaption: Conners Skalen zu Aufmerksamkeit und Verhalten-3. Version^40^, from which three subscale scores (*Inattention*, *Hyperactivity/Impulsivity*, *Executive Functions*) as well as the *ADHD Index* were computed. The ADHD Index was used as measure of interest in all analyses. It was developed for the study of pediatric samples in the age range of 6 – 18 years and is commonly used in psychological work with ADHD children^39,40^). Note that for visualization, the ADHD Index *t*-scores (instead of the ADHD Index sum scores) are shown as only *t*-scores allow for assignment of children into different verbally-labeled categories (see Results). Potential confounding effects of intelligence^41^ were controlled for by using individual SPM raw scores (see above) as covariate of no interest in all analyses.

### Experimental procedures

**Figure 1:**
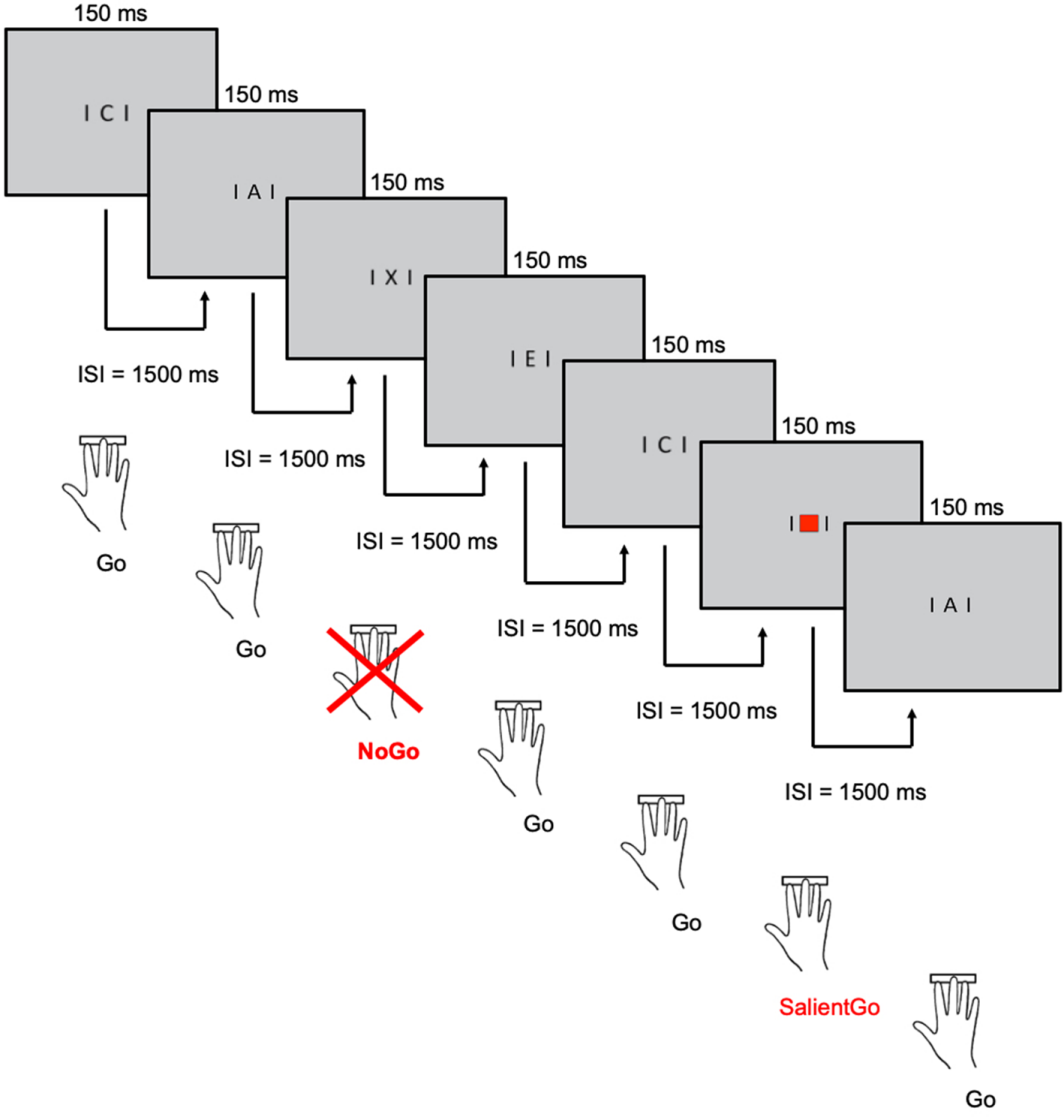
Schematic illustration of the Salience Continuous Performance Task (Salience CPT). Stimuli appeared on the screen for 150 ms followed by an inter-stimulus interval (ISI) of 1500 ms, summing up to a total trial duration of 1650 ms. Participants were instructed to press the space bar to all presented stimuli (i.e., letters and red squares) except the letter ‘X’.

The experimental paradigm used in the current study represents an adaptation of the classical Continuous Performance Task (CPT^18,19^). The CPT involves the continuous presentation of letter sequences, typically over the course of several experimental blocks. We adapted the ‘not X’ version of the classical CPT in which participants react via button press (space bar) to all presented letters (Go condition) except the letter X (NoGo condition)^18,19^. As we aimed to additionally assess neural markers of the processing of physical stimulus salience, a third condition was introduced in which a red square was presented at the center of the screen (SalientGo condition; see Fig. 1). We refer to this adapted version of the CPT as Salience CPT in the following. Participants were instructed to try to not get distracted by this salient event and to respond in the same way as to the Go condition (i.e., by pressing the space bar).

The task was presented on a computer in a soundproof cabin using PsychoPy (Version 2; https://www.psychopy.org/). The distance between child and screen was 120 cm. Black letters (size was matched to fit into a square of 10 × 10 cm) were presented on a light grey background (following the procedures of van Leeuwen et al.^42^). Each subject completed a practice session until the examiner was confident that the child fully understood the task. The task consisted of 800 trials (200 NoGo trials ‘X’; 520 StandardGo trials ‘letter other than X’; 80 SalientGo trials ‘red square’), presented in eight consecutive blocks (each with 100 stimuli; block duration 2 min 40 s). Between blocks, short breaks were introduced whose exact durations (max. 5 min) were determined by the children. The order of the stimuli within blocks was pseudo-randomized to ensure that each block consisted of 25% NoGo trials (‘X’) and 75% Go trials. Within each block, 10% of all trials were SalientGo trials, so that 13.33% of all Go trials were from the SalientGo condition (similar proportions were used before^43,44^). Further, it was ensured that at least two StandardGo trials were presented between two NoGo stimuli, between two SalientGo stimuli, and between NoGo and SalientGo stimuli.

Stimuli appeared on the screen for 150 ms followed by an inter-stimulus interval (ISI) of 1,500 ms, summing up to a total trial duration of 1650 ms (Fig. 1; Ref.^45^ for similar timing). All participants were instructed by the same experimenter (verbally and via written description on the computer screen) to press the space bar whenever a letter or a red square appeared on the screen, and to withhold their response when the letter ‘X’ was presented. In addition, children were advised to sit still and to avoid—as much as possible—any movements of their heads. Responses were registered between 200 and 1500 ms after stimulus onset only^44,46^. Trials in which no response was registered within this interval were treated as unanswered (MISS in Go trails and Correct Inhibitions in NoGo trails, see below). In total, the task took between 21:20 and 42:43 min depending on the individual-specific break durations (most participants made very short breaks and finished the task after 23–28 min), and was followed by a resting-state measurement for which separate instructions were provided after finishing the task (data not included in this report).

### Statistical analysis of behavioral measures

The Salience CPT generated multiple behavioral measures: Reaction times (RT) in correctly answered StandardGo trials (Standard HIT), RTs in correctly answered SalientGo trials (Salient HIT), the variability of these RT measures over time (computed as standard deviation across all trials of a condition), and finally, the percentage of commission and omission errors. The commission error ratio was calculated by dividing the number of false alarms (FA) by the total number of inhibition trials (FAs + Correct Inhibitions). Errors of omission occurred when children failed to press the space bar on Go trials, that is, when a letter other than ‘X’ or a red square appeared on the screen (MISS). The omission error ratio was calculated by dividing the number of missed trials by the total number of Go trials (i.e., MISS + HIT).

To test for associations between individual differences in ADHD Index and these behavioral parameters we used partial correlations (controlling for age, sex, and intelligence) after outliers were excluded (i.e., subjects with values > 3 *SD* above or below the mean of the respective variables of interest). Specifically, we used Spearman’s rank order correlation (*rho*) when the Shapiro–Wilk test indicates significant (*p* < 0.05) deviation of the data from normality and Pearson’s correlation (*r*) otherwise. For comparing different conditions (manipulation check) one-sample student *t*-tests were used after significant deviations of the data from normality were ruled out via the Shapiro–Wilk test (all *p* > 0.05). For extreme group comparisons (i.e., comparing highest and lowest third of the ADHD index distribution, control analyses) student independent samples *t*-tests were used after significant deviations of the data from normality were ruled out with the Shapiro–Wilk test (all *p* > 0.05) and significant deviations from homoskedasticity were ruled out with the Levene’s test (*p* > 0.05 in all cases).

### EEG recording and preprocessing

All pre-processing and analysis scripts can be found as reproducible scripts (jupyter notebooks^47^) at https://github.com/KirstenHilger/AttentionGo1.git.

EEG data were recorded with 64 active Ag/AgCl electrodes (arranged in an extended 10–20 layout) using an actiChamp amplifier (Brain Products GmbH, Gilching, Germany) and Fz as online reference electrode. Sampling rate was 1000 Hz. Impedance levels were kept below 10 kOhm, and an online low-pass filter with a high cutoff frequency was set to 280 Hz (notch filter off). Eye electrodes were put below the right eye (SO2) and lateral to both eyes (LO1, LO2). Two electrodes (M1, M2) were placed bilaterally on the mastoid bones.

Preprocessing and further analyses of EEG data were conducted in MNE Python^48^ (https://mne-tools.github.io/). At first, the data were re-referenced to average reference (excluding M1 and M2), down-sampled to 200 Hz, and band-pass filtered between 0.1 and 30 Hz. Eye movements and muscle artefacts were corrected via independent component analysis (ICA) using MNE’s ICA preprocessing tool (https://mne.tools/stable/generated/mne.preprocessing.ICA.html). FastICA was applied on the data which for this procedure were high-pass filtered at 8 Hz to guarantee data stationarity and thus improved convergence of the ICA decomposition. 60 ICA components were extracted for each subject. Eye movement and muscle artefact components were manually identified on six exemplary subjects through visual inspection and matching with typical EOG activity component maps^50^. For the rest of the participants, these artefact components were then identified via correlation (using MNE‘s corrmap tool^49^, https://mne.tools/stable/generated/mne.preprocessing.corrmap.html). Components automatically identified by this procedure were removed from the 0.1–30 Hz band-pass filtered data and the cleaned data were then re-referenced to average mastoids (as recommended in Luck^51^, and to reach comparability with previous literature on ADHD^35,36,52,53^). Subsequently, data were segmented into epochs of 1100 ms length time-locked to the onset of the stimulus (− 200 to 900 ms) and baseline-adjusted by subtracting the mean amplitude in the prestimulus period (− 200 to 0 ms) from all further data points. Finally, preprocessed data were manually screened to ensure high data quality. In one subject this revealed one single abnormal channel which was manually interpolated.

### Statistical analysis of neurophysiological measures

Event-related potentials (ERPs) were calculated for each subject by averaging over all trials within each condition, i.e., StandardGo, NoGo, and SalientGo. Incorrect trials representing omission errors in both Go conditions or commission errors in the NoGo condition were excluded. For statistical analyses of the inhibition-P3 (NoGo condition minus StandardGo condition), the salience-P3, and the salience-N2 (SalientGo condition minus StandardGo condition), signed area amplitudes and fractional area latencies (50%^51^) were calculated on time windows and electrodes that were determined via visual inspection of grand-average difference waves (as common in child ADHD research^54^). In brief, the inhibition-P3 was statistically analyzed in a time window between 300 and 600 ms at electrode POz, for the salience-P3 we extracted data from 350 to 600 ms at electrode POz, and the salience-N2 was examined on electrode Fz between 250 and 350 ms. Because we observed a rather posterior localization of the P3 component (which is however not unusual in pediatric populations^55,56^), we decided to extract also the signed area amplitude and fractional area latency values of the P3 on electrode Pz (in the same time windows) to allow for direct comparison with previous investigations^57^. Please see the respective parts of the results section for further details. Temporal variability of ERP components was operationalized as standard deviation (*SD*) of amplitude and latency measures over time.

Finally, associations between individual differences in ADHD Index and the strength (amplitude) and timing (latency) of the inhibition-P3, the salience-P3, and the salience-N2 as well as their variability were examined by calculating partial correlations while controlling for age, sex, and intelligence after exclusion of outliers (i.e., subjects with values > 3 *SD* above or below the mean of the respective variables of interest). Pearson’s correlation was used to compute the partial correlations except in cases where the Shapiro–Wilk test indicates significant (*p* < 0.05) deviation of the data from normality. In those cases Spearman’s rank order correlation (*rho*) was used. To protect against false positives resulting from multiple statistical tests, we corrected for the number of correlation tests conducted in relation to each ERP component (inhibition-P3, salience-N2, salience-P3). For each of these components, we tested for possible associations between the ADHD Index and the amplitude, latency, as well as the variability of these two parameters. Statistical significance was thus accepted at *p* < 0.0125 (i.e., *p* < 0.05/4, Bonferroni-corrected).

Statistical analyses were conducted in Python (Python 3.4, using the libraries SciPy, NumPy for statistical calculations, and Pandas for data organization), Matlab (Version 2020b; MathWorks, Inc., Natick, MA), JASP (version 0.13.1; https://jasp-stats.org/), and R (version 1.3.1056; www.r-project.org).

### Ethic statement

All study procedures were in accordance to the Declaration of Helsinki and approved by the ethics committee of the German Psychological Society (CG 062017) and the Ministry of Education and Cultural Affairs of the state of Baden-Württemberg. Informed written consent according to the Declaration of Helsinki and the guidelines of the German Psychological Society was obtained from all children and their parents, respectively.

## Results

### ADHD symptoms and behavioral performance

**Figure 2:**
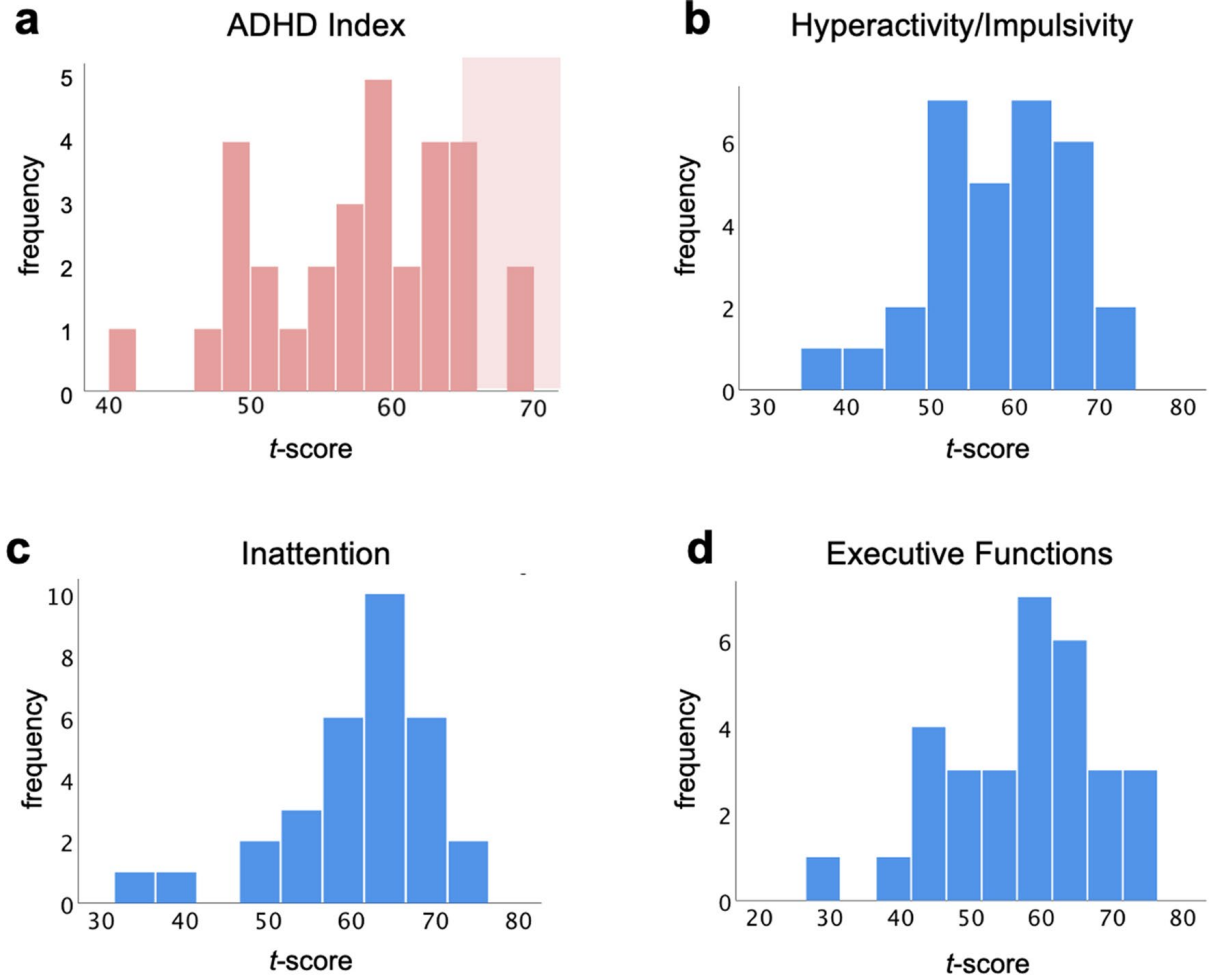
Distributions of participants’ scores on the Conners 3 Scales for Attention and Behavior. (**a)** Frequency histogram of Conners 3 ADHD Index *t*-scores. *t*-scores ≥ 65 are interpreted as elevated scores^39^ and marked by a light red shading in the background. (**b**–**d**) Frequency histograms of *t*-scores of Conners 3 sub-scales for Inattention, Hyperactivity/Impulsivity, and Executive Functions, respectively.

Descriptive statistics of the ADHD Index (Table 1, Fig. 2) indicate substantial variation in our sample, supporting its use as a valuable instrument for assessing between-person variation in attentional problems and executive functioning also in the subclinical range. Frequency distributions of ADHD Index *t*-scores are depicted in Fig. 2a. 19 out of 31 children exhibited ADHD *t*-scores < 60, which is interpreted as ‘average—typical levels of concern’. Nine children had a *t*-score ≥ 60 but < 65, i.e., ‘high average score—slightly more concerns than typically reported’, and the remaining three children had *t*-scores ≥ 65 but < 70, i.e., ‘elevated score—more concerns than typically reported’. Specifically, two of these three children had an ADHD Index *t-*score of 69, and one participant had a *t*-score of 65 (this latter case is not illustrated as separate bar in Fig. 2a). ‘Very elevated’ scores (ADHD Index *t*-score ≥ 70) indicating ‘many more concerns than typically reported’ were not observed in our sample (verbal interpretations of ADHD Index scores^39^). For the sake of completeness, Fig. 2b–d also show the distribution of participants’ scores on the subscales Hyperactivity/Impulsivity, Inattention, and Executive Functions. These are however not used in the analyses reported in the following.

**Table 1:** Descriptive statistics of the Conners 3 scales and behavioral performance in the Salience Continuous Performance Task (Salience CPT).

	*M*	*SD*	*Median*	*Min*	*Max*
**Conners 3 scales**					
ADHD index	5.32	4.36	4	0	14
Inattention	13.71	6.01	13	1	28
Hyperactivity/Impulsivity	12.42	7.64	12	0	31
Executive Functions	10.13	6.00	9	0	23
**Salience CPT**					
RT (StandardGo, HIT)	390.36	77.82	393.96	232.88	551.48
RT (SalienceGo, HIT)	423.10	91.67	433.62	233.42	575.92
Var RT (StandardGo, HIT)	160.21	39.23	154.13	80.45	255.54
Var RT (SalienceGo, HIT)	162.95	48.32	155.07	89.13	265.52
Omission error ratio (Go, MISS)	0.04	0.05	0.02	0.00	0.23
Commission error ratio (NoGo, FA)	0.41	0.18	0.41	0.06	0.76

RT, mean reaction time (in ms) across all trials of a condition; Var RT, intra-subject variability (standard deviation) in reaction times (in ms) across all trials of a condition; *M*, mean (across subjects); *SD*, standard deviation (across subjects); *Min*, minimum value observed across all participants; *Max*, maximum value observed across all participants.

Descriptive statistics of performance measures in the Salience CPT task are also listed in Table 1. The ratio of omission errors (see Methods) in the StandardGo and Salience Go conditions combined (4%) and false positives in the NoGo condition (commission errors; 41%) was comparable to previous research in subclinical pediatric samples^18^ and reaction times varied between subjects within a plausible range. Participants needed more time to react to SalientGo compared to StandardGo trials (*t*(30) = 5.991, *p* < 0.001, ${\stackrel{-}{M}}_{Diff}$ = 0.033 s, 95%-Confidence Interval (CI): 0.022 s to 0.044 s, Cohens *d* = 1.076). No significant correlations were observed between the ADHD Index and behavioral markers of response inhibition (commission errors: *r* = 0.15, *p* = 0.456) or salience processing (mean RT in SalienceGo HIT trials: *r* = − 0.04, *p* = 0.830; standard deviation of RT in SalienceGo HIT trials: *r* = 0.04, *p* = 0.825; salience effect in RTs, i.e., mean RT in SalienceGo HIT trials—mean RT in StandardGo HIT trials: *r* = − 0.20; *p* = 0.280). Note that we also found no such associations a) when testing for differences between extreme groups, i.e., upper vs. lower third of the ADHD Index distribution (commission errors: *t*(18) = 1.081, *p* = 0.294 , 95%-CI: − 0.414 to 1.367, Cohens *d* = 0.483; mean RT in SalienceGo HIT trials: *t*(18) =  − 0.714, *p* = 0.485, ${\stackrel{-}{M}}_{Diff}$ = 0.033 s, 95%-CI: − 1.197 s to 0.568 s, Cohens *d* = − 0.319; standard deviation of RT in SalienceGo HIT trials: *t*(18) =  − 0.632, *p* = 0.535, ${\stackrel{-}{M}}_{Diff}$ = 0.024 s, 95%-CI: − 1.160 s to 0.602 s, Cohens *d* = − 0.283; salience effect in RTs: mean RT in SalienceGo HIT trials—mean RT in StandardGo HIT trails: *t*(18) =  − 0.876, *p* = 0.393, ${\stackrel{-}{M}}_{Diff}$ = 0.012 s, 95%-CI: − 1.272 s to 0.499 s, Cohens *d* = 0.393), b) when repeating the correlation analyses with the three subscales of the ADHD Index (all − 0.33 < *r* < 0.11; all *p* > 0.092), or c) when testing for logarithmic, quadratic, or cubic relationships (all *R*^*2*^ < 0.15; all *p* > 0.211). For completeness, we tested also for associations between ADHD Index and the mean RT in StandardGo HIT trials (*r* = 0.01, *p* = 0.955), the standard deviation of RT in StandardGo HIT trials (*rho* = 0.12, *p* = 0.55), and the omission error ratio (*rho* = 0.12, *p* = 0.533).

### Event-related brain potentials

**Figure 3:**
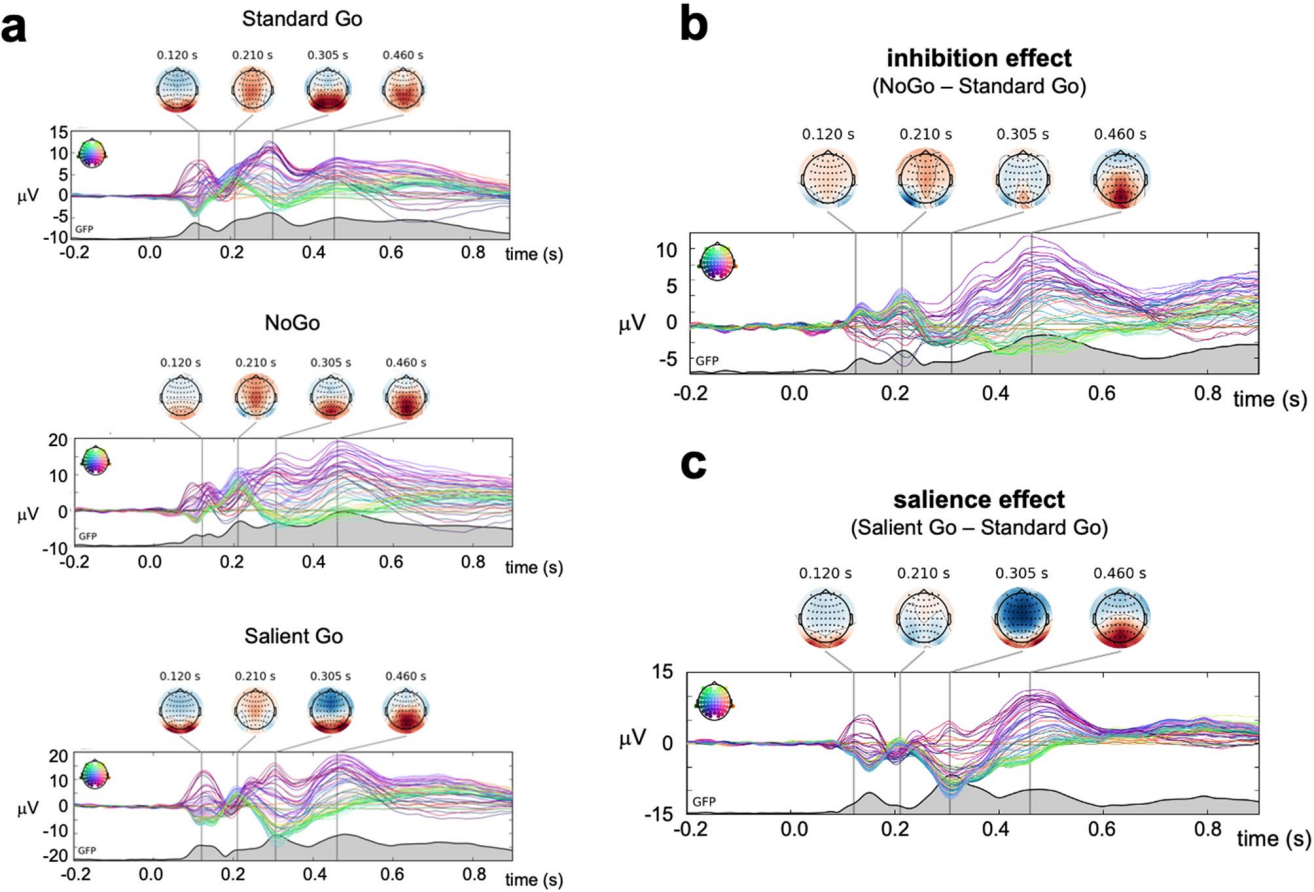
Grand-average ERPs at all EEG channels. Different channels are plotted in different colors, according to the color inset in the left upper corner of each plot. The global field power is shown at the bottom of each plot as a black line. All scalp topographic maps are scaled from –15 µV to + 15 µV. (**a**) Grand-average ERPs to all three conditions, shown separately. (**b**) Grand-average ERP difference waves representing the group-averaged effect of inhibition (NoGo – StandardGo). (**c**) Grand-average ERP difference waves representing the group-averaged effect of salience processing (SalientGo – StandardGo).

Grand-average ERPs for all three conditions (StandardGo, NoGo, SalientGo) are shown in Fig. 3a. Descriptively, all experimental conditions elicited a visually evoked P1 at around 100 ms with a distribution typical for linked mastoid references, i.e., maximum positive amplitude over lateral occipital scalp^58,59^, indicating selective attention towards the presented stimuli^59,60^. Especially in the NoGo condition, a further positivity became visible at around 200 ms over anterior scalp sites, which might reflect a P2 component. A noticeable negativity over frontal electrodes was observed at around 300 ms, especially in the SalientGo condition, which we interpret as N2^31^. Following this effect, a marked positivity developed between 300 and 700 ms with a localization over central and posterior electrodes as expected for the P3. Note that such a late timing of the P3 component is not unusual and seems to be typical for pediatric samples in comparable paradigms^56,57,61^.

### P3 and N2 responses to inhibition and salience processing

**Figure 4:**
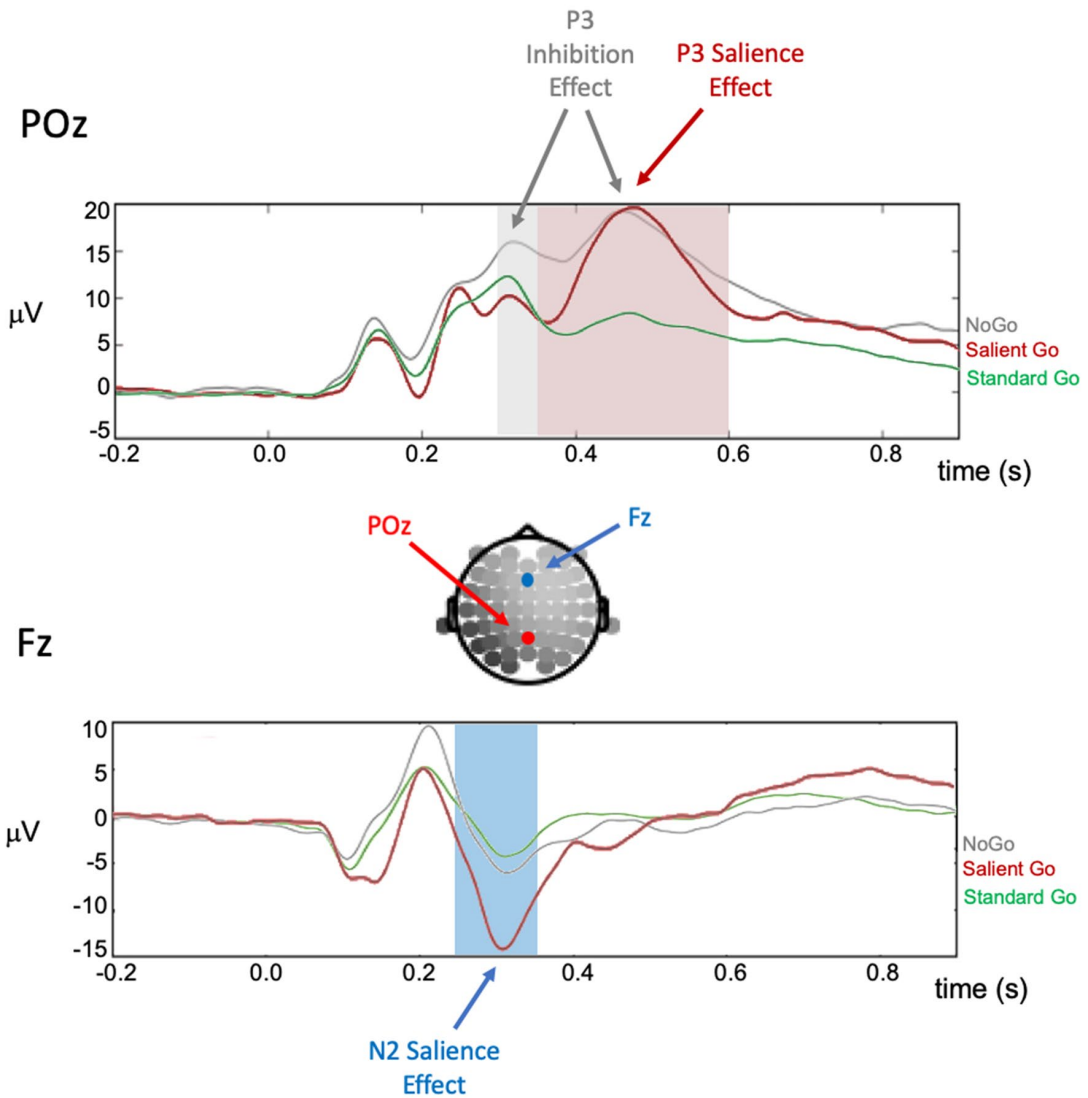
Grand-average ERPs at electrode POz and Fz for all three conditions. In the scalp map, POz location is highlighted in red (P3 effects), Fz location is highlighted in light blue (N2 effects). Time windows used in all analyses are highlighted in grey for the inhibition-P3 (i.e., 300 – 600 ms), in light red for the salience-P3 (i.e., 350 – 600 ms), and in light blue for the analyses of the salience-related N2 component (i.e., 250 – 350 ms).

The neural signature of inhibitory processes (inhibition effect: NoGo – StandardGo; see Fig. 3b and Fig. 4) became most visible in the form of an increased P3 component (greater positivity) at posterior electrodes centered around POz that started at around 300 ms and reached its maximum at 460 ms after stimulus presentation. We therefore used POz as electrode of interest for statistical analyses. The time window for calculating inhibition-related amplitude and latency measures was defined between 300 and 600 ms to best capture this component in our sample (similar time windows in ADHD research have been used in comparable studies^56,61^. Within this time window, the difference between NoGo and StandardGo ERPs was significantly different from zero (signed area amplitude; one-sample two-tailed *t*-test: *t*(30) = 16.87, *p* =  < 0.001, ${\stackrel{-}{M}}_{Diff}$ = 919 μVs, 95%-CI: 801–1020 μV).

Salience processing (SalientGo – StandardGo) also elicited an enhanced (i.e., more positive-going) P3 component (Figs. 3c, 4). This P3 effect was also most visible at electrode POz, started at around 350 ms, and reached its maximum at 455 ms post stimulus onset. Therefore, POz was again defined as electrode of interest and salience-related amplitude and latency measures were derived between 350 and 600 ms. Within this time window, the difference between Salient and StandardGo ERPs was significantly different from zero (signed area amplitude; one-sample two-tailed *t*-test: *t*(30) = 17.30, *p* =  < 0.001, ${\stackrel{-}{M}}_{Diff}$ = 799 μVs, 95%-CI: 705 μVs to 894 μVs). The saliency-dependent N2 effect became visible as fronto-central negativity at ~ 300 ms and was analyzed at Fz in a time window of 250 – 350 ms (see Fig. 4; similar timing of N2 in pediatric samples has been reported in previous studies^61^; signed area amplitude; one-sample two-tailed *t*-test: *t*(30) = 14.98, *p* < 0.001, ${\stackrel{-}{M}}_{Diff}$ = 78.3 μVs, 95%-CI: 67.6 μVs to 88.9 μVs.

### Association between ADHD symptoms and neurophysiological markers of inhibition and salience processing

The main aim of the current study was to examine the association between continuous variations in ADHD symptoms (as indicated by the ADHD Index score) and neuronal markers of inhibition and salience processing. These correlations were tested on the electrodes and time windows identified in the previous section, and a corrected significance threshold of *p* < 0.0125 was applied as described in more detail in the Methods section. All correlation results are summarized in Table 2.

**Table 2:** ADHD Index and neuronal markers of inhibition and salience processing measured at POz (and Pz) for inhibition and salience effects in P3 and at Fz for salience effects in N2. Significant associations (*p* < .05) are depicted in bold letters.

	*r*_*part*_*/rho*_*part*_	*p*_*part*_
**Mean effects**		
*Inhibition-P3*		
Amplitude	−.34 (−.26)	.078 (.182)
Latency	**.38** (.28)	**.043** (.139)
*Salience-P3*		
Amplitude	−.**44** (−.33)	.002* (.096)
Latency	.28 (.17)	.148 (.384)
*Salience-N2*		
Amplitude	−.02	.921
Latency	−.09	.667
**Temporal variability**		
*Inhibition-P3*		
Amplitude	−.**51 (**−.**41)**	.005* (.029)
Latency	.11 (.13)	.584 (.515)
*Salience-P3*		
Amplitude	−.**51 (**−.**41)**	.006* (.033)
Latency	**.46 (.38)**	**.014 (.045)**
*Salience-N2*		
Amplitude	.08	.688
Latency	.22	.267

Associations passing the Bonferroni corrected-threshold of *p* = .0125 (four comparisons) are marked with an asterisk. *r*_*part*_, Pearson’s correlation coefficient for the partial correlation controlling for effects of age, sex, and SPM raw scores; *rho*_*part*_, Spearman’s rank order correlation coefficient for the partial correlation controlling for effects of age, sex, and SPM raw scores (used when data violates significantly the assumption of normality. This refers to six associations: Between ADHD Index and mean effects in Salience-P3 amplitude at POz and Pz, between ADHD Index and mean effects in Salience-N2 amplitude at Fz, between ADHD Index and temporal variability in Salience-P3 amplitude at POz and Pz, and between ADHD Index and temporal variability in Salience-N2 amplitude), *p*_*part*_, *p* value of significance for the partial correlation.

**Figure 5:**
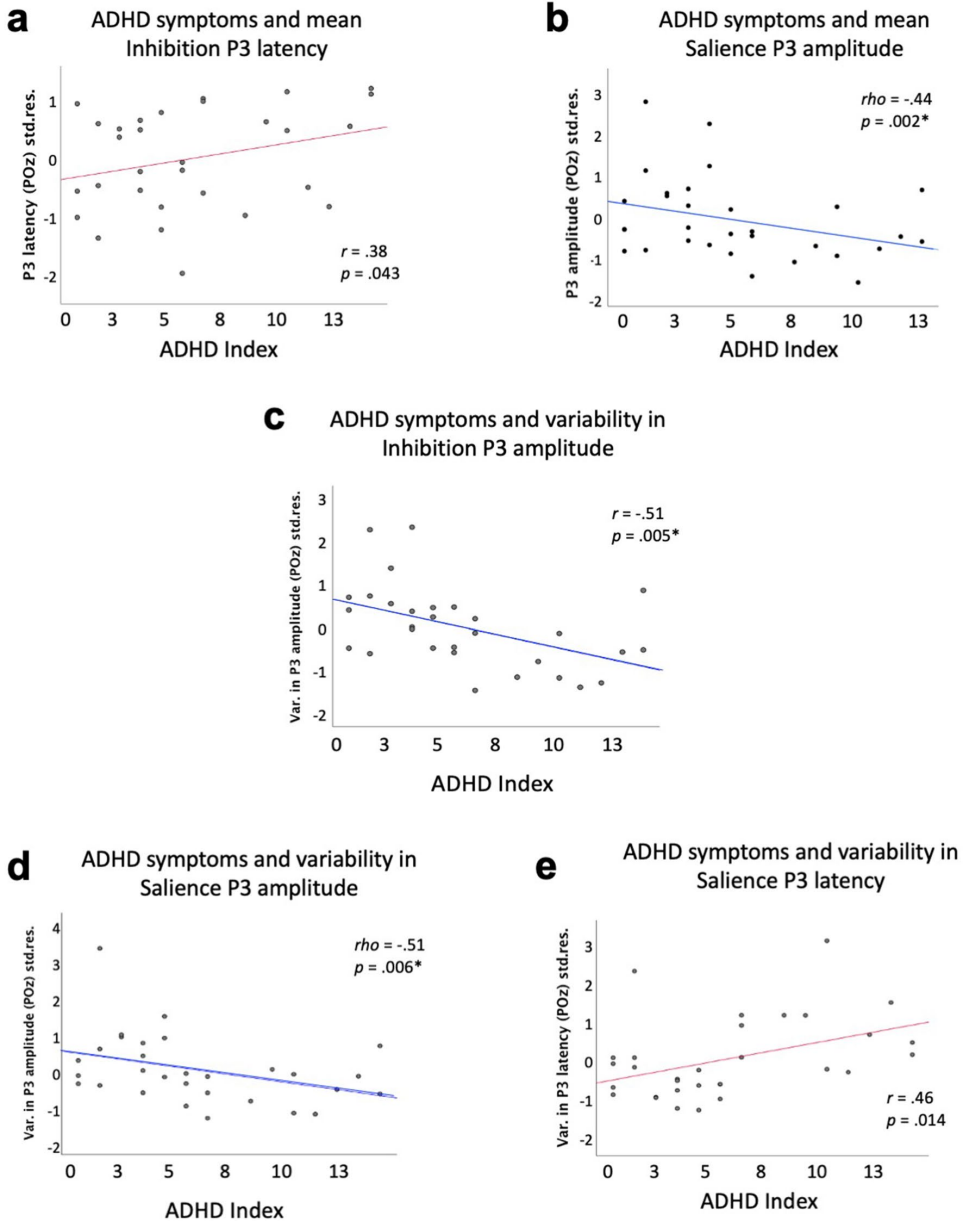
Scatterplots for significant associations between ADHD Index and neuronal markers of inhibition and salience processing, after controlling for effects of age, sex, and general intelligence. For illustration of partial correlations, the *y*-axis depicts the standardized residuals resulting from linear regression of age, sex, and general intelligence (SPM raw score) on the neural marker of interest (i.e., the signed area amplitude or fractional area latency; see Methods). (**a**) Higher ADHD Index is descriptively associated with increased latencies of the inhibition-related P3. (**b**) Higher ADHD Index is significantly associated with higher amplitude of the salience-related P3. (**c**) Higher ADHD Index is significantly associated with less variability in the amplitude of the inhibition-related P3. (**d**) Higher ADHD Index is significantly associated with less variability in the amplitude of the salience-related P3. (**e)** Trend-level support for an association between higher ADHD Index and higher variability in the latency of the salience-related P3. Associations passing the Bonferroni corrected-threshold of *p* = .0125 (four comparisons) are marked with an asterisk. *r*_*.*_, partial Pearson’s correlation coefficient for the partial correlation controlling for effects of age, sex, and SPM raw scores; *rho*_,_, partial Spearman’s rank order correlation coefficient for the partial correlation controlling for effects of age, sex, and SPM raw scores (used when data violates significantly the assumption of normality). Std.res., standardized residuals; Var., variance.

We observed no relationship between inhibition-related P3 amplitude and ADHD Index (Hypothesis 1a; *r* = − 0.34; *p* = 0.078), but a trend towards longer latencies of the inhibition-P3 in children with more parent-reported ADHD symptoms (Hypothesis 1b; *r* = 0.38; *p* = 0.043; Fig. 5a). In respect to the salience-related P3 effect (exploratory analyses), we found a significant association between more ADHD symptoms and smaller P3 amplitude (*rho* = − 0.44; *p* = 0.002; Fig. 5b), but no effect on P3 latency (*r* = 0.28; *p* = 0.148 2). Next, we assessed possible associations with the variability of these neuronal responses over time. Contrary to our Hypothesis 2a, we observed significantly less variability in the amplitude of the inhibition-P3 in children with more ADHD symptoms (*rho* = − 0.51; *p* = 0.005; Fig. 5c) and no effect on the latency of the inhibition-P3 (Hypothesis 2b; *r* = 0.11; *p* = 0.584). Our results furthermore revealed that children with more ADHD symptoms exhibited significantly less variability in the amplitude of the salience-P3 (*r* = − 0.51; *p* = 0.006; Fig. 5d) and a trend towards more fluctuations in the latency of this component (*r* = 0.46; *p* = 0.014; Fig. 5e). For comparability with previous research, we repeated all P3-related analyses at Pz which yielded similar results (see Table 2 for direct comparison).

Finally, we tested for associations between ADHD Index and salience-related N2 effects. No significant correlations were observed between the ADHD Index and mean amplitudes (*rho* = − 0.02; *p* = 0.921), latencies (*r* = − 0.09; *p* = 0.667), or the variability of the salience-related N2 (variability in amplitudes: *rho* = 0.08, *p* = 0.688; variability in latencies: *r* = 0.22, *p* = 0.267).

## Discussion

In the current study, we observed significant associations between parent-reported problems in attention and executive functioning and neural mechanisms of inhibition and salience processing in a subclinical sample of school-aged children. Although alterations in the inhibition-related P3 component are one of the most established neurophysiological markers of clinical ADHD^21,22^, we observed in this pediatric community sample no correlative association with the amplitude of the inhibition P3 amplitude and only trend-level support for longer inhibition P3 latencies in children with more ADHD symptoms. However, we found that ADHD-related behavioral symptoms are significantly associated with decreased P3 amplitudes in response to physical stimulus salience and with reduced between-trial variance in the amplitude of both inhibition- and salience-related P3 effects. The P3 related to physical stimulus salience and the temporal variability of ERPs associated with executive control functions may thus be valuable continuous indicators of ADHD-related executive control processes.

### ADHD and neurophysiological correlates of inhibitory processes

Deficits in response inhibition have been identified as one of the most crucial deficits in ADHD, and the investigation of respective event-related potentials (ERPs) provides insights into spatial and temporal characteristics of neural mechanisms underlying inhibitory control processes^17^. In the behavioral performance data, individual differences in inhibition (as indexed by the number of commission errors) showed a positive but non-significant relationship with parent-reported ADHD symptoms. The absence of a reliable correlation is, however, not due to a lack of variability in the behavioral outcome measure, as the ADHD Index showed substantial between-person differences.

In contrast to our Hypothesis 1, we observed no attenuation of the amplitude of the inhibition-P3 in children with higher levels of reported ADHD symptoms and only trend-level support for longer latencies of the inhibition-P3. Reduced P3 amplitudes in ADHD have been interpreted as indicator of diminished capabilities to evaluate and process the target stimulus and in general as reflecting a lower degree of process control during response inhibition^62^. The absence of this effect in our community sample suggest that such evaluative processes are not fundamentally impaired in typically-developing children with more problems in attention and executive functioning. Delayed P3 latencies in ADHD, on the other hand, are interpreted as indicator of an inappropriately late allocation of attention^63^, which may further hinder the fast and successful inhibition of the dominant behavioral response. Our trend-level results concerning the P3 latency is not incompatible with this assumption, but provides no robust basis for evaluating it either. However, as our sample of children did neither show the expected association in behavior nor exhibit obvious behavioral problems at school, we speculate that also children with relatively higher ADHD Index scores are able to compensate any existent neurophysiological predispositions for attentional and executive problems^64^. In contrast, when neurophysiological alterations exceed a critical level, compensation would fail and observable behavioral problems may emerge—making a diagnosis of ADHD more likely. However, our observation that the ten children with the highest ADHD Index (including those four children with ADHD diagnosis) did not differ in their behavior from those ten children with the lowest ADHD Index lends no direct support for this interpretation and indicates a need for further empirical investigation of potential compensatory mechanisms.

### ADHD and neurophysiological markers of salience processing

Although much neuroimaging research on ADHD has focused on the salience network and the identification of neural correlates of increased distractibility in children with ADHD^24,25^ and even though ERP studies revealed associations between ADHD and the processing of motivational or emotional salience^27,29^, potential relations between ADHD and electrophysiological indices of ‘pure’ physical stimulus salience have only rarely been investigated in previous EEG research. One previous investigation demonstrated that it is quite hard to disentangle pure effects of stimulus salience from effects of novelty (indexed by the early-developing anterior novelty P3^65^), targetness^30^, or effects of stimulus deviance (as typically investigated in oddball paradigms^63^). In our ‘Salience Continuous Performance Task’ (Salience CPT) paradigm, the ‘X’ is rare (a deviant) but *not* physically more salient than the other stimuli, while the red square is rare (also a deviant) *and* physically salient. Also, it requires the same response as the StandardGo condition (and thus does not differ in its ‘targetness’), and both deviant stimuli (i.e., the ‘X’ and the red square) are identical throughout the entire experiment, so that their neurophysiological correlates do not reflect novelty responses. Therefore, our experimental task allows to isolate cognitive processes related to the processing of a perceptually salient (and thus potentially distracting) but task-irrelevant stimulus—and may thus be well-suited for investigations of the mechanisms underlying the increased distractibility in children with ADHD.

We observed no associations with the ADHD Index at the behavioral level and no relation with salience-P3 latencies, but significantly reduced salience-P3 amplitudes in children with higher ADHD Index scores. The posterior salience-associated P3 component is suggested to evolve from cingulate and temporo-parietal brain regions and to reflect the ascribed importance of a stimulus^66,67^. Godefroid and Wiersema^30^ observed ADHD-related effects in the salience-P3 of opposite direction (i.e., higher amplitudes in ADHD), but only in response to the novelty of the stimuli and not in response to their targetness or deviancy. Thus, our result do not contradict their findings but provide important insights into the importance of further differentiation between different aspects of saliency, i.e., between mechanisms of novelty, targetness, deviancy, and physical salience, and their contributions to the increased distractibility in ADHD. Note, that the later timing of the salience-P3 in contrast to the inhibition-P3 (Fig. 4) is not surprising. In contrast to the novelty P3 that typically shows reduced but earlier-peaking positive deflections (in comparison with the inhibition-P3^68^), the salience-related P3 deflections are typically later-developing and characterized by a steeper increase^68^.

The absence of any relationship between ADHD Index scores and the N2 amplitude or latency converges with results from a previous group-comparison study that found no significant N2 differences between adults with ADHD and healthy controls^30^. Although the N2 has frequently been investigated in response to NoGo stimuli in both adult and child samples with ADHD^46,69^(for a meta-analysis of NoGo N2 effects see Ref.^70^), respective findings are hardly comparable with ours, as in most of these studies the N2-elicting stimulus was both salient and task-relevant (i.e., the NoGo stimulus). Processes related to response inhibition vs. handling task-irrelevant physical salience can thus not be clearly dissociated on these grounds. To sum up, our study introduces a new variant of the CPT paradigm that allows to investigate relatively ‘pure’ effects of physical stimulus salience, different from more motivational-behavioral salience effects of novelty or targetness, and our results suggest that ADHD-related behaviors are associated with the amplitude of P3 effects elicited by stimulus salience. However, as our sample size was small, the reported effects can only be treated as explorative and hypothesis-generating, requiring further empirical investigation.

### Neural variability as additional index of problems in attention and executive functioning

In contrast to the rather selective results for correlative associations between ADHD Index and mean amplitudes and mean latencies, associations between ADHD Index and the temporal variability of ERP components were markedly present—even in our comparably small sample. However, in contrast to Hypothesis 2, we observed a negative association between amplitude variability and ADHD Index (less variability in the inhibition-related P3 amplitude in children with higher ADHD Index), and no association in respect to the variability of inhibition-P3 latencies.

Our hypothesis concerning inhibition-P3 amplitude variability was based on two previous investigations^35,36^. However, even though these studies provide initial evidence in favor of an association between ADHD symptoms and ERP amplitude variability, these two studies differed markedly from the present investigation in numerous aspects: Both studies a) investigated clinical samples and, in the case of Gonen-Yaacovi et al.^35^ studied adult ADHD patients, b) applied different experimental paradigms during EEG acquisition (a visuo-spatial backmasking task interpreted as working memory task^36^; an auditory choice reaction time task and a visual Go-Nogo Task^35^, and, most crucially, c) both studies operationalized ERP variability in different ways, i.e., different from each other and different from the current study. Gonen-Yaacovi et al.^35^ assessed neural variability of a different EEG component, i.e., of the sensory-evoked P100 and N100 components (time window: 80 – 150 ms, P100 measured at occipital and N100 at central electrodes), while Myatchin et al.^36^ assessed global ERP amplitude variability, i.e., pooled across scalp electrodes and across target and non-target stimuli, and within a broad time window of 0 – 600 ms. We thus do not necessarily consider our results of a negative correlation between inhibition-related P3 amplitudes and ADHD Index as incompatible with these previous data or with the general hypothesis that ADHD symptoms may be related to maladaptive patterns of ERP amplitudes over time. Rather, we conclude that further research is needed to specify the circumstances under which positive or negative associations with ADHD symptoms arise.

Finally, we also explored potential associations between ADHD symptoms and the variability of the P3 and N2 components associated with salience processing. Similar as for the inhibition-P3, we observed significantly less variability in the amplitudes in children with higher ADHD Index, but no significant effects concerning the latency of this component or in respect to the salience-N2. Based on these finding, we propose that the focus of ADHD studies that was previously primarily on tasks requiring some form of response inhibition and on mean ERP components, should be broadened to additionally consider salience-related processes and especially the temporal variability in related neural mechanisms.

### Towards dimensional models of ADHD

A more dimensional conceptualization of ADHD was recently proposed^10^. Empirical research supporting this proposal demonstrated continuous covariations between ADHD symptoms in healthy individuals on the one hand and behavioral^11,13^ or neural^16^ characteristics on the other hand. Importantly, all neurophysiological associations reported in the present study were also observed across a continuous and broad range of individual variations. In our subclinical sample, we found trend-level support for one effect that is a relatively established finding in ADHD group-comparison research, i.e., longer inhibition-P3 latencies^21,22^, and interesting associations with ERPs evoked by the processing of stimulus salience. We therefore propose that some of the neural characteristics that have previously been identified as biomarkers of ADHD may reflect more general mechanisms linked to individual variations in behavior and cognition that are only in their extremes ‘defined’ as ADHD.

### Limitations and future directions

As already stated in the Methods section, the sample size of 31 children (which was restricted by pragmatic reasons) limits the ability of the study to detect effects of weak or moderate effect size. We therefore stress that such results (correlations < 0.48) can only be treated as hypothesis-generating in the present study, but may serve to inform future studies with larger sample sizes. Also, we assessed rather subtle variations in a dimensional marker within a subclinical sample, which may also call for a replication in larger samples to ensure sufficient statistical power. Obvious candidates for such future research are the robust correlations observed between ADHD Index and two P3 variability measures. We propose that future studies should also consider variability measures rather than exclusively focusing on mean (trial-average) amplitude and latency effects.

Also, we cannot fully rule out potential selection biases when registering for the present study. Due to the a-priori restriction to specific types of secondary schools, our sample was not representative of the entire population of school-aged children, and parents who believed that their child may have problems with attention or executive functions may have been more willing to participate in and support respective research. However, as this circumstance may have broadened the range of the ADHD Index distribution, this might be a strength rather than a weakness of the current study.

Further, our study relied on only one integrated questionnaire measure to estimate the level of ADHD-related behaviors, i.e., the ADHD Index rated by the children’s parents. As it has been shown that the estimates of ADHD symptoms can vary substantially between different scales and more or less objective sources of information (children vs. parents vs. teachers^71^), combining multiple ratings from different sources would be preferable in future studies to provide more robust estimates of behavioral variation. Finally, by adopting a cross-sectional approach, our results do not allow to draw any conclusions in respect to the question of whether the observed neurophysiological signatures of ADHD symptoms will persist into adulthood, or whether they may rather reflect a delay in cortical development that can potentially be overcome (delayed-maturation hypothesis^72^, dimensional approach Ref.^73^). Our results suggest that future longitudinal research should also take into account the temporal variability of neurophysiological measures.

## Conclusion

Our study investigated established neurophysiological markers of ADHD symptoms in a subclinical community sample of school children. Unlike previous group comparison studies with clinical ADHD samples, we found no evidence for amplitude reductions of the inhibition-P3 in relation to ADHD-related behaviors, but provide trend-level support for a delay of inhibition-related P3 latencies. However, we identified the temporal variability of inhibition-P3 amplitudes as a possible correlate of ADHD. Our study introduced a new version of the classical Continuous Performance Task that allows to simultaneously assess, in addition to effects of response inhibition, also effects of physical stimulus salience, and our results suggests temporal variability of the amplitude of the salience-related P3 as additional marker of ADHD. We extend previous group-comparison research to the continuous range of subclinical variations and show that some neurophysiological indicators of clinical ADHD may represent more general mechanisms of attention and executive functioning. This supports recent proposals of a more dimensional conceptualization of ADHD, according to which the diagnosis of ADHD would represent an extreme end of continuous variations not only in attention and executive functioning but also in the underlying neural processes.

## Data Availability

All analysis code used in the current study has been deposited on GitHub at https://github.com/KirstenHilger/AttentionGo1. The data of the whole project will be deposit on zenodo.org after the project is completed (part of ongoing longitudinal project, see below). The data used in the actual study can be assessed from the authors upon direct request.
